# Publisher Correction: Stress granules plug and stabilize damaged endolysosomal membranes

**DOI:** 10.1038/s41586-023-06882-z

**Published:** 2023-11-22

**Authors:** Claudio Bussi, Agustín Mangiarotti, Christian Vanhille-Campos, Beren Aylan, Enrica Pellegrino, Natalia Athanasiadi, Antony Fearns, Angela Rodgers, Titus M. Franzmann, Anđela Šarić, Rumiana Dimova, Maximiliano G. Gutierrez

**Affiliations:** 1 The Francis Crick Institute, London, UK; 2 Max Planck Institute of Colloids and Interfaces, Potsdam, Germany; 3 Institute of Science and Technology Austria, Klosterneuburg, Austria; 4 Department of Physics and Astronomy, Institute for the Physics of Living Systems, University College London, London, UK; 5 Center for Molecular and Cellular Bioengineering, Biotechnology Center, Technische Universität Dresden, Dresden, Germany

**Keywords:** Membrane biophysics, Organelles

Correction to: *Nature* 10.1038/s41586-023-06726-w Published online 15 November 2023

In the version of the article initially published, there was an error in Fig. 1c where the *y*-axis label now reading “GAL-3 area/cell area” originally said “G3BP1 area/cell area”. In Fig. 4h, two labels now reading “ZNFX1” said “ZNFZ1”. The errors have been corrected in the HTML and PDF versions of the article.

