# Supplementary material for: Stress granules plug and stabilize damaged endolysosomal membranes
Source: Nature. 2023 Nov 15;623(7989):1062–9. doi: 10.1038/s41586-023-06726-w (PMC10686833; doi:10.1038/s41586-023-06726-w)

nature portfolio

https://doi.org/10.1038/s41586-023-06726-w

Supplementary information

Stress granules plug and stabilize damaged endolysosomal membranes

In the format provided by the authors and unedited

Stress granules plug and stabilise damaged endolysosomal membranes

Claudio Bussi[1*], Agustín Mangiarotti[2], Christian Vanhille-Campos[3], Beren Aylan[1#], Enrica Pellegrino[1#], Natalia Athanasiadi[1], Antony Fearns[1], Angela Rodgers[1], Titus M. Franzmann[4], Anđela Šarić[3], Rumiana Dimova[2] and Maximiliano G. Gutierrez[1*]

1- The Francis Crick Institute, London, United Kingdom, 2- Max Planck Institute of Colloids and Interfaces, Potsdam, Germany. 3- Institute of Science and Technology Austria, Klosterneuburg, Austria. 4- Center for Molecular and Cellular Bioengineering, Biotechnology Center, Technische Universität Dresden, Dresden, Germany.

*Corresponding author, #equal contribution

Supplementary information Guide

Supplementary Methods on Molecular dynamics	Pages 2-3
Supplementary table 1	Page 4
Legends to Supplementary videos 1-15	Pages 5-6
Supplementary Fig. 1 Uncropped western blot images	Pages 7-11

Supplementary Methods

Molecular Dynamics

In order to elucidate the physical mechanisms underlying the plugging effect of stress granules (SG) observed *in vivo* and *in vitro* upon membrane poration we performed Molecular Dynamics (MD) simulations of model lipid vesicles embedded in a protein-rich medium undergoing controlled poration. We consider a system consisting of a model lipid vesicle[1] separating the simulation box in two compartments with different particle compositions. The membrane is made of particles of size σ (the MD unit of length) interacting according to parameters $\epsilon = 4.34\ k_{\mathrm{B}}T, r_{\mathrm{m}} = 1.12\ \sigma, \zeta = 4, \mu = 3, \theta = 0, r_{\mathrm{c}} = 2.6\ \sigma$, following the original model notation[1]. To model the different conditions of the endosomal lumen and cytoplasmic protein solution we consider two different types of particles inside and outside of the vesicle (particles labelled as Solute and Protein respectively). All particles in the system have size σ and interact with one another and with the membrane via a cosine-squared potential with a Weeks-Chandler-Andersen (WCA) repulsive tail (see Equation 1)[2]. This type of potential can create gas, liquid or crystalline phases depending on the strength of the interaction ϵ and the cutoff distance r_{c} (note for example that for $r_{\mathrm{c}} = r_0$ only the repulsive part of the potential remains) which makes it well suited to capture the different behaviour of the protein solution in the cytoplasm and the endosomal lumen. As shown in the table below, most interactions are purely repulsive to model the dissolved phases of the inner and outer lumen (Solute-Solute and Protein-Protein) respectively and to prevent mixing through a closed membrane (Solute-Membrane). To explore the effects of condensate formation upon mixing of the two solutions and how membrane wetting by the condensate droplets affects the process, we introduce two parameters, ϵ_{LLPS} and ϵ_{wet}, that control the Solute-Protein and the Protein-Membrane interactions (see table below). In this way, depending on the value of ϵ_{LLPS}, protein solution particles will phase-separate into liquid droplets when mixed with lumen particles ($\epsilon_{\mathrm{LLPS}} = 1.5\ k_{\mathrm{B}}T$) or remain dissolved ($\epsilon_{\mathrm{LLPS}} = 0.0\ k_{\mathrm{B}}T$). Similarly, by varying the strength of the interaction between protein and membrane particles ($\epsilon_{\mathrm{wet}} < 1\ k_{\mathrm{B}}T$), we can explicitly control the wetting capacity of the droplets. Finally, note that if $\epsilon_{\mathrm{LLPS}} = 0\ k_{\mathrm{B}}T$ or $\epsilon_{\mathrm{wet}} = 0\ k_{\mathrm{B}}T$ then we set $\epsilon = 1\ k_{\mathrm{B}}T, r_{\mathrm{c}} = r_0, \alpha = 0$ for the corresponding interactions to preserve the repulsive part of the potential. Otherwise, $\alpha = 1$ and $\epsilon = \epsilon_{\mathrm{LLPS}}$ or $\epsilon = \epsilon_{\mathrm{wet}}$. In Equation 1 we define the interaction potential, and we present the specific parameters controlling the interaction between the different particles in the table below.

Equation 1: Particle interaction potential. Weeks-Chandler-Andersen term for steric interactions and cosine/squared term for attraction

$$E(r) = E_{\mathrm{WCA}}(r) + E_{\mathrm{C2}}(r)$$

$$E_{\mathrm{WCA}}(r) = \epsilon\left[\left(\frac{r_0}{r}\right)^{12} - 2\left(\frac{r_0}{r}\right)^{6} + 1\right]\ r < r_0$$

$$E_{\mathrm{C2}}(r) = \begin{cases} -\alpha\epsilon & r < r_0 \\ -\alpha\epsilon\cos\left(\dfrac{\pi(r - r_0)}{2(r_{\mathrm{c}} - r_0)}\right)^2 & r_0 \le r < r_{\mathrm{c}} \\ 0 & r \ge r_{\mathrm{c}} \end{cases}$$

We performed MD simulations of this system for different degrees of poration on the vesicle (different pore radii r_{p}) and different ranges of protein-solute and protein-membrane interactions ($\epsilon_{\mathrm{LLPS}}, \epsilon_{\mathrm{wet}}$) to explore the effects droplet formation and wetting have on mixing through the membrane pore. Initially, our model membrane consists of $N_{\mathrm{mem}} = 4322$ particles, each representing a bilayer portion of around 10 nm in size ($\sigma = 10$ nm), which results in a vesicle of ~400 nm in diameter. After relaxation, we

introduce solute and protein particles in the system at different volume fractions ($p_{\mathrm{in}} = 0.7, p_{\mathrm{out}} = 0.4$, resulting in a hypoosmotic shock) and artificially create a pore of radius $r_{\mathrm{p}} \in [80,140]$ nm by removing a portion of the membrane. Note that a hypoosmotic shock is required to put the vesicle under tension stress and inhibit spontaneous sealing of the pore. In this setup, we then let the system evolve under different conditions for droplet formation (controlled by ϵ_{LLPS}) and wetting (controlled by ϵ_{wet}). Further, in order to explore the effects of protein concentration, we systematically varied the amount of protein particles in the outer lumen while keeping a constant packing fraction (thus maintaining the osmotic conditions) by replacing a fraction of these particles by non-interacting solute particles. We evolve our system following Langevin dynamics (damping factor of 1τ, where τ is the MD unit of time) at constant volume and constant total number of particles. To integrate the system, we used a timestep of $\mathrm{d}t = 0.01\tau$. For each set of parameters $\{r_{\mathrm{p}}, \epsilon_{\mathrm{LLPS}}, \epsilon_{\mathrm{wet}}\}$ we simulate $N_{\mathrm{rep}} = 10$ replicas to obtain statistically relevant results. All simulations were performed using the LAMMPS Molecular Dynamics package[3].

References

1 Yuan, H., Huang, C., Li, J., Lykotrafitis, G. & Zhang, S. One-particle-thick, solvent-free, coarse-grained model for biological and biomimetic fluid membranes. *Phys Rev E Stat Nonlin Soft Matter Phys* **82**, 011905, doi:10.1103/PhysRevE.82.011905 (2010).

2 Cooke, I. R., Kremer, K. & Deserno, M. Tunable generic model for fluid bilayer membranes. *Physical Review E* **72**, 011506, doi:10.1103/PhysRevE.72.011506 (2005).

3 Thompson, A. P. *et al.* LAMMPS - a flexible simulation tool for particle-based materials modeling at the atomic, meso, and continuum scales. *Computer Physics Communications* **271**, 108171, doi:https://doi.org/10.1016/j.cpc.2021.108171 (2022).

Supplementary Table 1- Information on the interaction parameters following *Equation 1*

Interacting pair	$\epsilon\ [k_{\mathrm{B}}T]$	$r_0\ [\sigma]$	$r_{\mathrm{c}}\ [\sigma]$	α
Solute – Solute	1	1	1	0
Solute – Protein	ϵ_{LLPS}	1	2.5	1
Solute – Membrane	1	1.5	1.5	0
Protein – Protein	1	1	1	0
Protein – Membrane	ϵ_{wet}	1.5	3.75	1

Supplementary Videos:

Video S1. Live-cell imaging sequence showing the stress granules response of iPSDM expressing G3BP1-GFP (magenta) and GAL-3-RFP (green) treated with 1mM of LLOMe. Time frame = 20 s. Images are z-stack maximum intensity projections. Video related to Fig. 1a.

Video S2. Live-cell imaging sequence showing the stress granules response of iPSDM expressing G3BP1-GFP (magenta) and GAL-3-RFP (green) treated with 1mM of LLOMe. Time frame = 20 s. Images are z-stack maximum intensity projections. Video related to Fig. 1a.

Videos S3-S7. Zoom-in live-cell imaging examples from the videos shown before (Videos S1 and S2) highlighting G3BP1-GAL-3 interactions. Arrowheads illustrate the polarised distribution of G3BP1 (plug pattern) and how the condensates and membrane damage recognition by GAL-3 simultaneously increases over time. Videos related to Fig. 1a.

Video S8. Live-cell imaging sequence showing the stress granules response of iPSDM expressing G3BP1-GFP (magenta) and GAL-3-RFP (green) treated with 1mM of LLOMe. Time frame = 1 s. Images show one z-section of 150nm. Scale bar: 2μm. Video related to Fig. 1h.

Video S9. G3BP1/RNA localized condensate formation upon vesicle poration. DOPC:DOPS (9:1) giant unilamellar vesicle (labelled with 0.1% mol ATTO 647N-DOPE, shown in green) enclosing a solution of GFP-G3BP1 (magenta) at pH=7.5 (see Methods). The external pH is quickly lowered to pH=5 using a microfluidic device, and a hypotonic solution containing poly(A)-RNA is included to promote poration (details in Methods). G3BP1/RNA condensation is observed at the pore region, stabilizing the membrane damage. Note that the condensate appears at different positions over time because the vesicle rotates due to the externally applied mild flow, needed to keep the GUV in place. The flow also presses the vesicle against the microfluidic posts and deforms it during poration. The time stamps are given in minutes:seconds (mm:ss). The acquisition speed is 0.650 s/frame. The field of view is 41 x 41 μm.

Video S10. Partial Z-stack sequence of the damaged GUV as shown in Fig. 2c,d. The field of view is 33.5 x 33.5 μm.

Video S11. Glycinin localized condensate formation after vesicle poration. DOPC giant unilamellar vesicle (labelled with 0.1% mol ATTO 647N-DOPE, cyan) enclosing a solution of the protein glycinin (containing 4 % FITC labelled glycinin, yellow) at pH = 7.4. The external pH is quickly lowered to pH=4.8 using a microfluidic device, and a hypotonic solution is included to promote poration. Glycinin condensation is observed at the pore region, stabilizing the membrane damage. Note that the condensate appears at different positions over time because the vesicle rotates due to the externally applied mild flow, needed to keep the GUV in place. The flow also presses the vesicle against the microfluidic posts (in the upper left corner of the sequence) and deforms it during poration. The time stamps are given in minutes:seconds (mm:ss). The acquisition speed is 0.650 s/frame. The field of view is 66.7 x 66.7 μm.

Video S12. Simulation of a damaged vesicle with liquid-like interactions between protein and solute particles and a wetting-like attraction between protein and membrane particles (green curve in Fig. 2i). We observe plugging at the pore and engulfment of the growing droplet.

Video S13. Simulation of a damaged vesicle with liquid-like interactions between protein and solute particles (pink curve in Fig. 2i). We observe plugging at the pore as a condensate droplet forms.

Video S14. Simulation of a damaged vesicle with no attractive interactions between protein and solute particles (grey curve in Fig. 2i). No condensation occurs and the two solutions mix freely through the pore.

Video S15. Representative example of a LysoTracker leakage/recovery assat using U2OS WT and G3BP DKO cells. The movie shows a frame with cells before the addition of LLOMe (indicated as pre-LLOMe), 2min after (indicated as LLOMe), and a time lapse sequence after LLOMe removal and a washout step (indicated as washout). The sequence shows the lack of lysosomal recovery in U2OS G3BP DKO cells, and it also highlights two LysoTracker-positive structures in U2OS G3BP DKO cells (black circles) that lose the LysoTracker probe (red circles) during the washout step (suggestive of continuous leakage and lack of efficient repair).

Blots related to Figure 4b

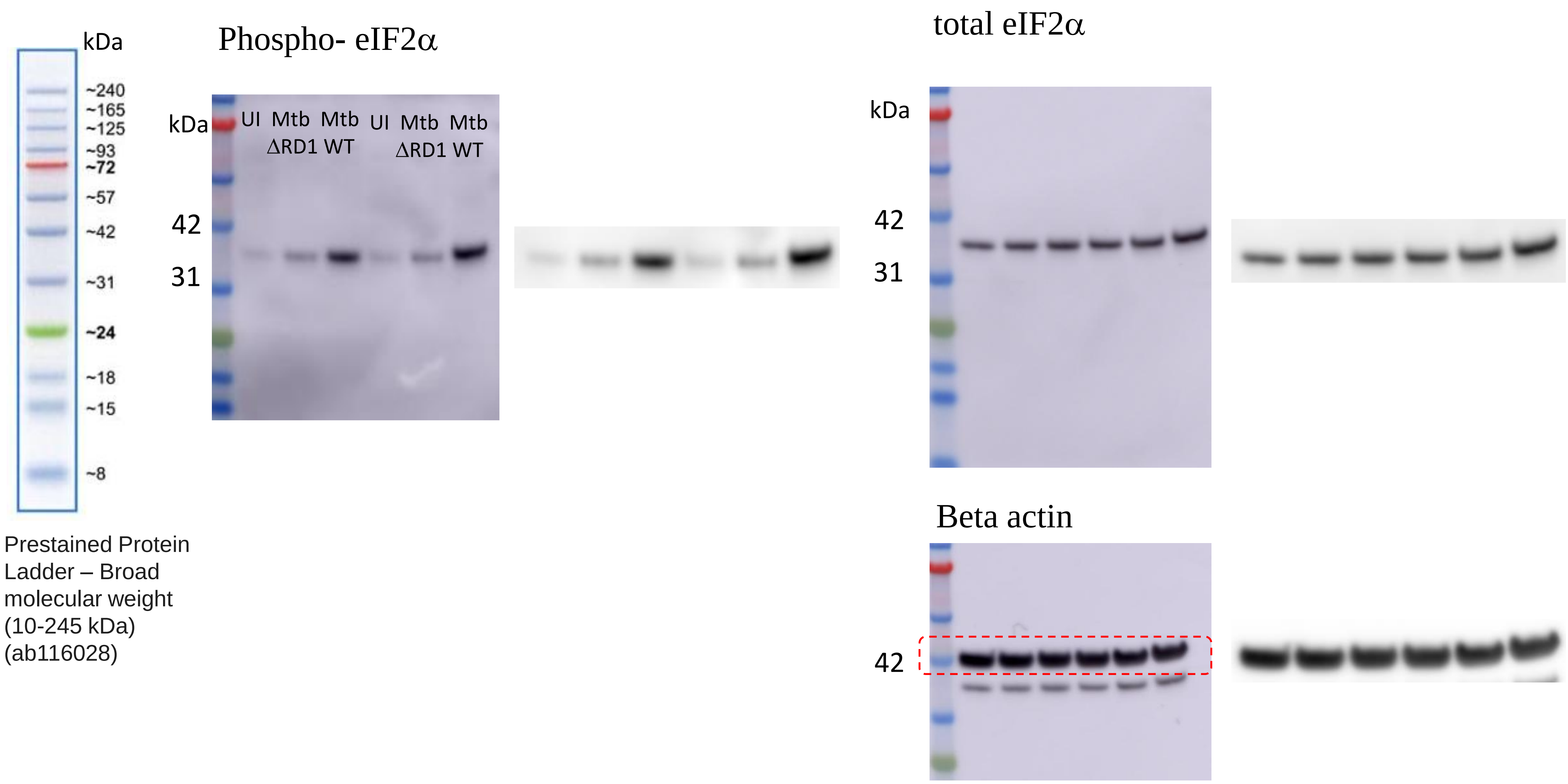

Blots related to Extended Data 2c

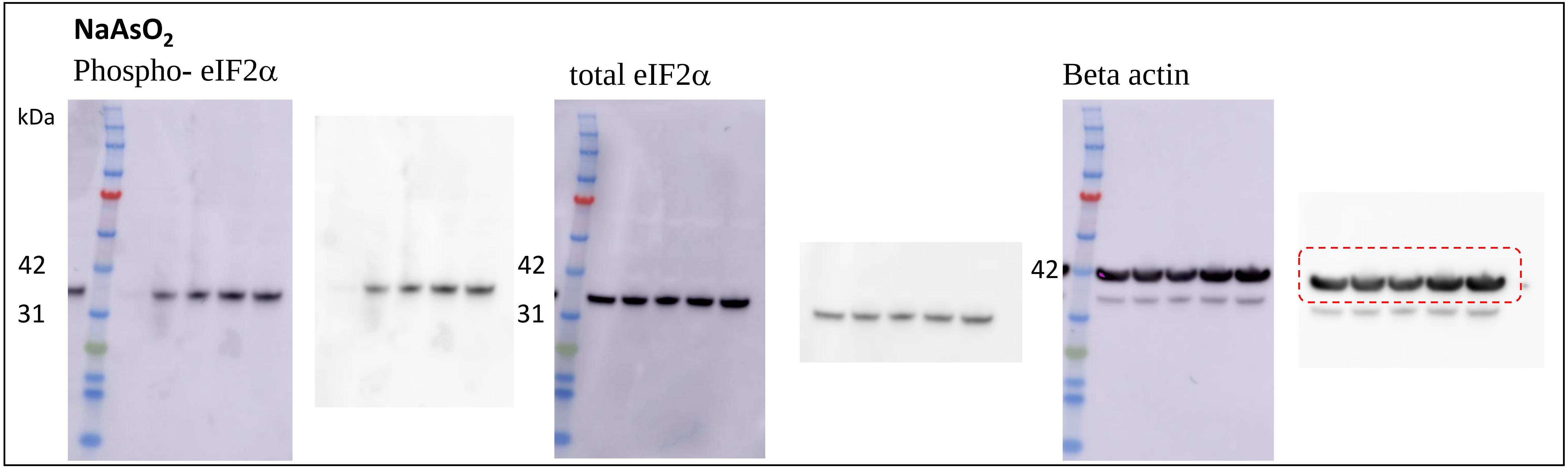

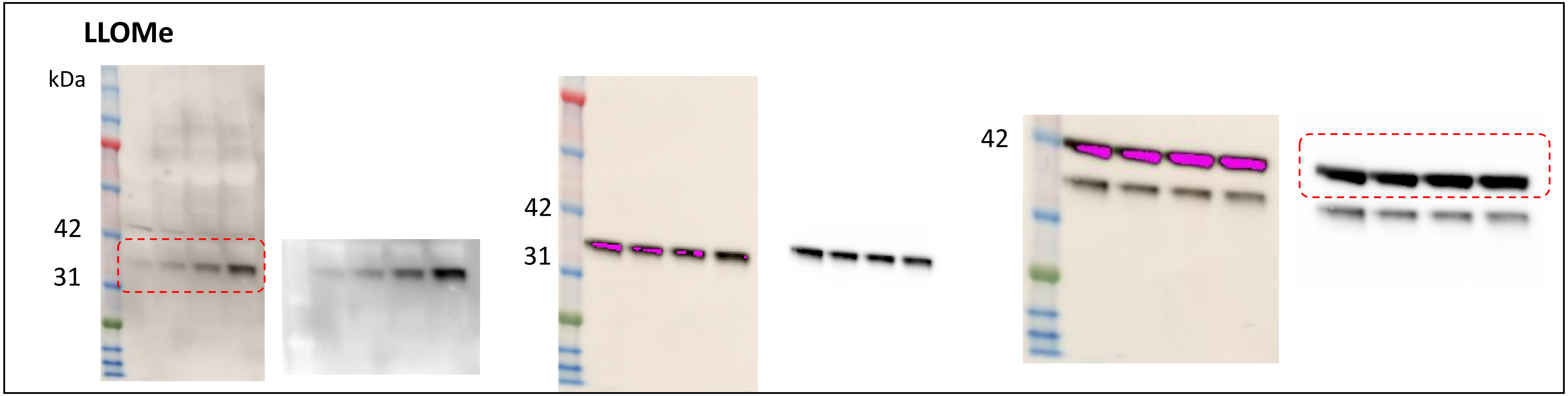

Blots related to Extended Data 6b,h,i

Extended Data 6b
(iPSDM)

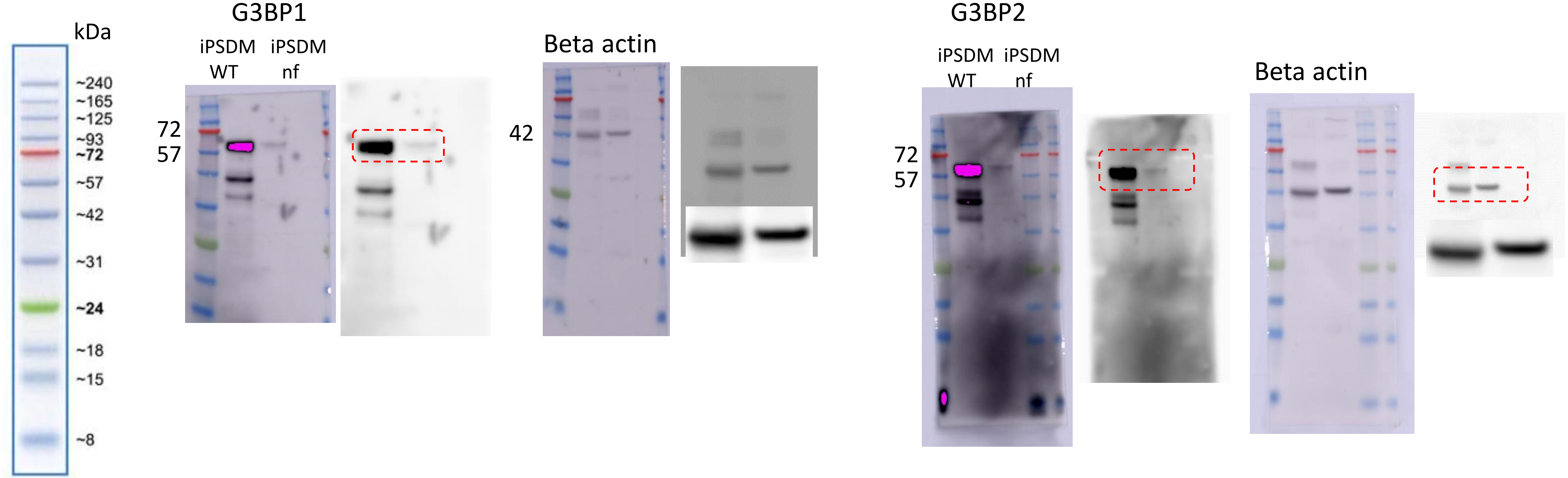

Prestained Protein Ladder – Broad molecular weight (10-245 kDa) (ab116028)

Extended Data 6h
(HeLa cells)

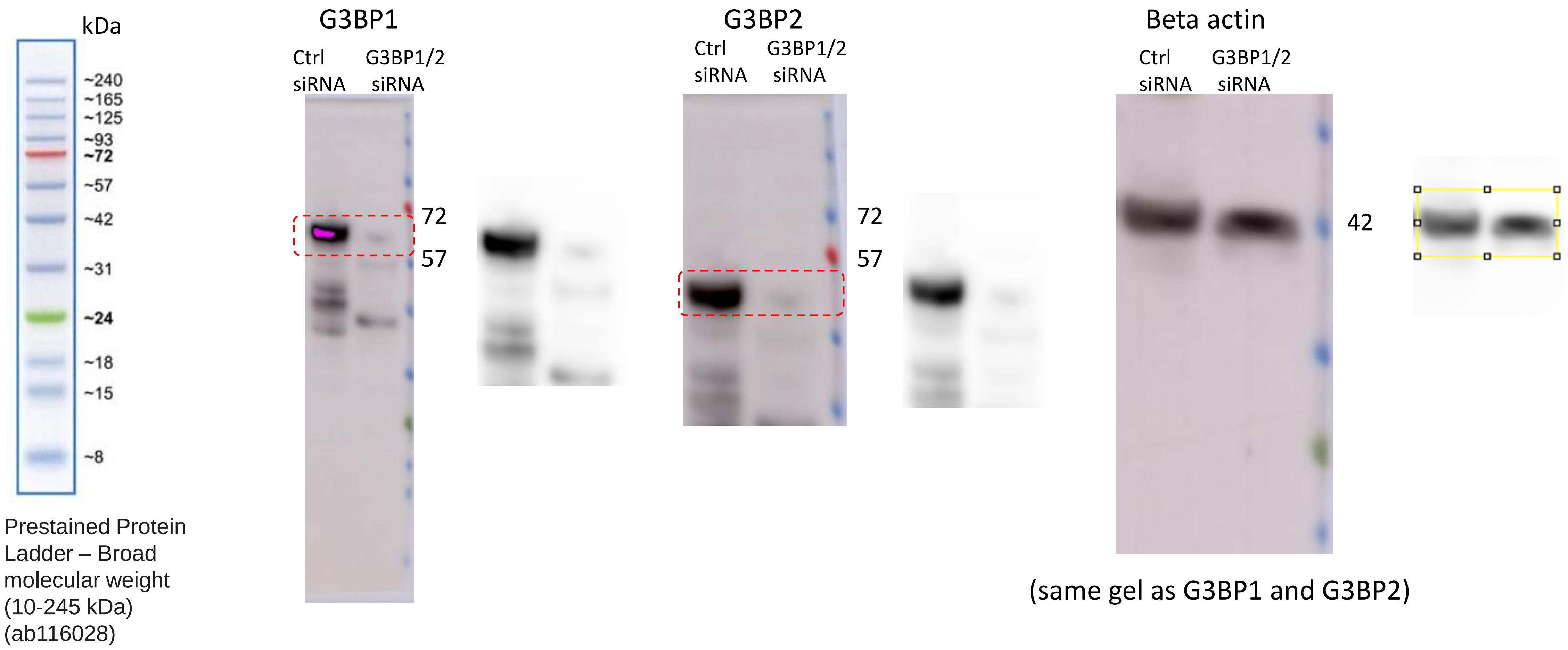

Extended Data 6i (hMDM)

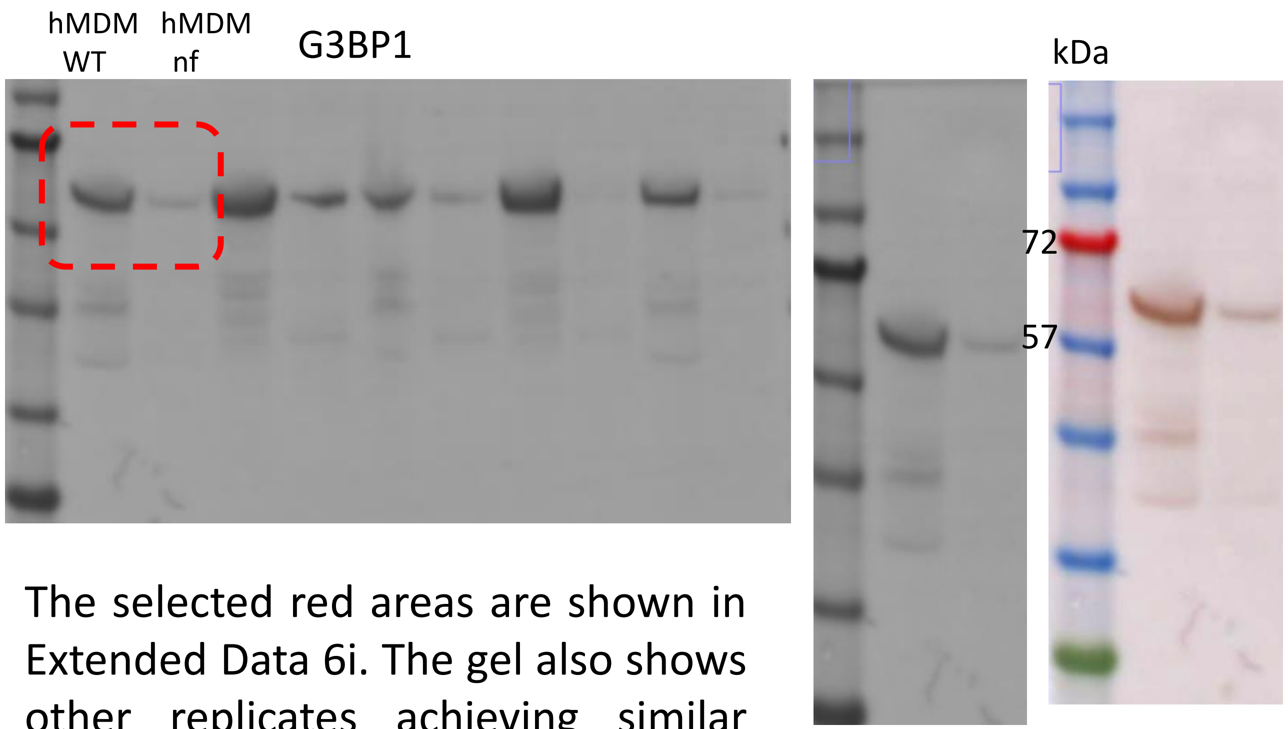

The selected red areas are shown in Extended Data 6i. The gel also shows other replicates achieving similar results (1st line WT, 2nd line nucleofected cells).

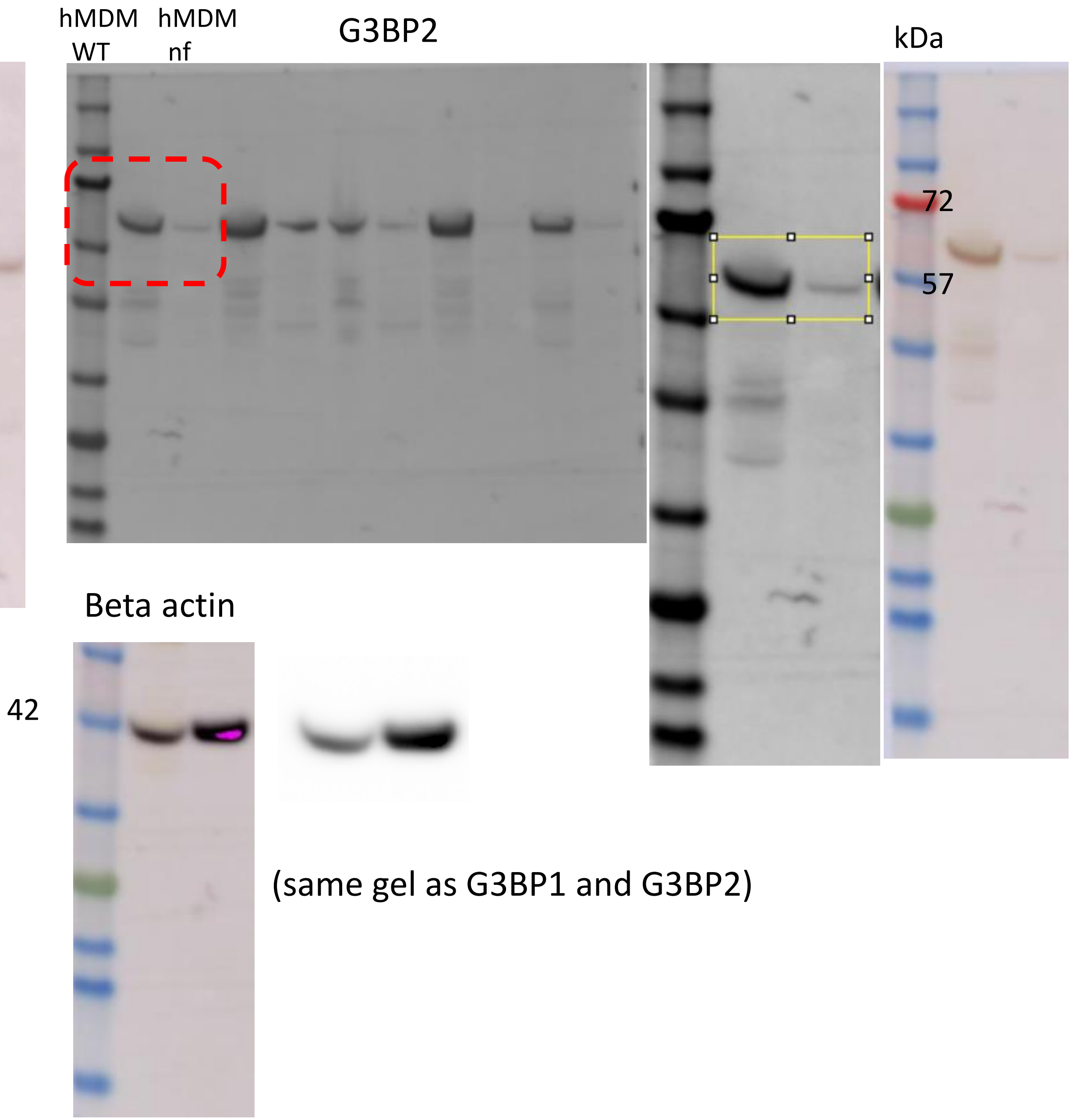

Blots related to Extended Data 7c

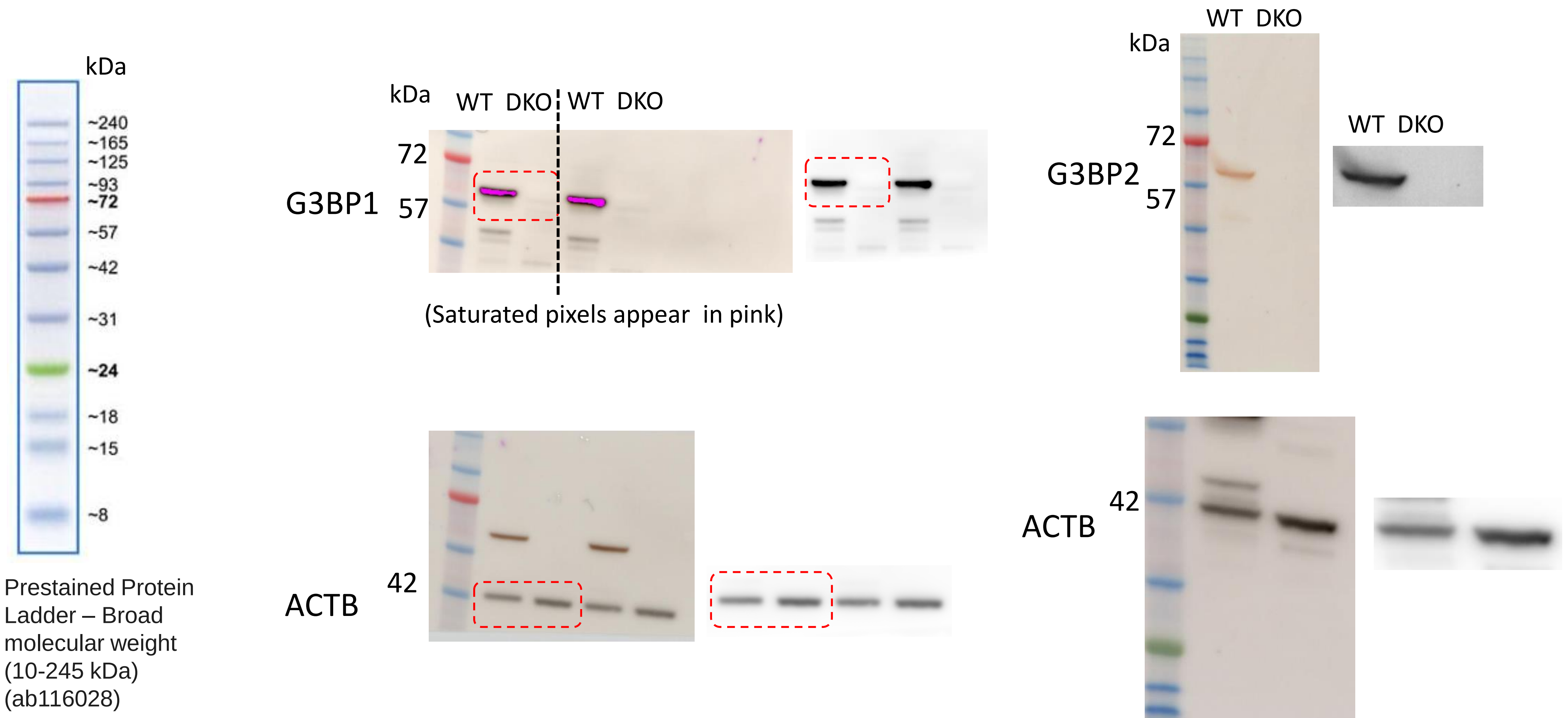

Supplement: Supplementary file 1 — This file contains Supplementary Methods on Molecular dynamics, Supplementary table 1, Legends to Supplementary videos 1-15 and Supplementary References. [file 41586_2023_6726_MOESM1_ESM.pdf]
