# Supplementary material for: Stress granules plug and stabilize damaged endolysosomal membranes
Source: Nature. 2023 Nov 15;623(7989):1062–9. doi: 10.1038/s41586-023-06726-w (PMC10686833; doi:10.1038/s41586-023-06726-w)
Supplement: Supplementary file 2 — Reporting Summary [file 41586_2023_6726_MOESM2_ESM.pdf]

nature portfolio

Corresponding author(s): Claudio Bussi and Maximiliano G. Gutierrez

Last updated by author(s): Oct 3, 2023

Reporting Summary

Nature Portfolio wishes to improve the reproducibility of the work that we publish. This form provides structure for consistency and transparency in reporting. For further information on Nature Portfolio policies, see our Editorial Policies and the Editorial Policy Checklist.

Statistics

For all statistical analyses, confirm that the following items are present in the figure legend, table legend, main text, or Methods section.

n/a	Confirmed	
☐	☒	The exact sample size (*n*) for each experimental group/condition, given as a discrete number and unit of measurement
☐	☒	A statement on whether measurements were taken from distinct samples or whether the same sample was measured repeatedly
☐	☒	The statistical test(s) used AND whether they are one- or two-sided<br>*Only common tests should be described solely by name; describe more complex techniques in the Methods section.*
☒	☐	A description of all covariates tested
☐	☒	A description of any assumptions or corrections, such as tests of normality and adjustment for multiple comparisons
☐	☒	A full description of the statistical parameters including central tendency (e.g. means) or other basic estimates (e.g. regression coefficient) AND variation (e.g. standard deviation) or associated estimates of uncertainty (e.g. confidence intervals)
☐	☒	For null hypothesis testing, the test statistic (e.g. *F*, *t*, *r*) with confidence intervals, effect sizes, degrees of freedom and *P* value noted<br>*Give P values as exact values whenever suitable.*
☒	☐	For Bayesian analysis, information on the choice of priors and Markov chain Monte Carlo settings
☒	☐	For hierarchical and complex designs, identification of the appropriate level for tests and full reporting of outcomes
☒	☐	Estimates of effect sizes (e.g. Cohen's *d*, Pearson's *r*), indicating how they were calculated

Our web collection on statistics for biologists contains articles on many of the points above.

Software and code

Policy information about availability of computer code

Data collection	VT-iSIM images were acquired using Olympus cellSens software. High-content images were acquired using Harmony 4.9 (Perkin Elmer). Western Blot membranes were obtained using an Amersham Imager 680 instrument (GE Life Sciences).
Data analysis	Image processing of confocal, VT-iSIM microscopy images and western blots: FIJl/lmageJ (version 2.1.0/1.53t). Image processing and deconvolution of VT-iSIM images were done using Huygens Essential software (Scientific Volume Imaging B.V, Netherlands, v 21.1). Statistical analysis was performed using Graph Pad Prism 10 software or R Studio 2023.03.0 (R 4.2.2). High-content imaging analysis was done using Harmony 4.9 (Perkin Elmer) and mean values were obtained using Harmony 4.9 or R 4.2.2. The number of biological replicates and the statistical analysis performed and post hoc tests used can be found in the figure legends. RNA-seq heatmaps were done using Morpheus (https://software.broadinstitute.org/morpheus/). Spatial point pattern analysis was done using spatstat package in R (version 3.0-6) as specified in the methods section. Molecular dynamics analysis are specified in detail in the methods section. Custom code is available at https://github.com/Saric-Group and https://github.com/cvanhille/SGporecondensation

For manuscripts utilizing custom algorithms or software that are central to the research but not yet described in published literature, software must be made available to editors and reviewers. We strongly encourage code deposition in a community repository (e.g. GitHub). See the Nature Portfolio guidelines for submitting code & software for further information.

March 2021

Data

Policy information about availability of data

All manuscripts must include a data availability statement. This statement should provide the following information, where applicable:

- Accession codes, unique identifiers, or web links for publicly available datasets
- A description of any restrictions on data availability
- For clinical datasets or third party data, please ensure that the statement adheres to our policy

Data availability: The data needed to evaluate the conclusions of the study are present in the manuscript or in the supplementary materials. Source data for the main and Extended Data figures are provided with this paper. Source data for gels and blots are provided as Supplementary information (Supplementary Figure 1). Source Data are provided for Figs. 1 to 4 and Extended Data 1 to 10. Code availability: Custom analysis codes were used to extract pore lifetime and solution exchange measurements from simulations. All analysis code used is available on a public GitHub repository (see data analysis and methods section).

Human research participants

Policy information about studies involving human research participants and Sex and Gender in Research.

Reporting on sex and gender	N/A
Population characteristics	N/A
Recruitment	N/A
Ethics oversight	N/A

Note that full information on the approval of the study protocol must also be provided in the manuscript.

Field-specific reporting

Please select the one below that is the best fit for your research. If you are not sure, read the appropriate sections before making your selection.

☒ Life sciences ☐ Behavioural & social sciences ☐ Ecological, evolutionary & environmental sciences

For a reference copy of the document with all sections, see nature.com/documents/nr-reporting-summary-flat.pdf

Life sciences study design

All studies must disclose on these points even when the disclosure is negative.

Sample size	No statistical method was used to predetermine sample size. Standard considerations based on expected variations from previous experiments [10.1126/science.aat9689, 10.15252/embj.2020104494] were applied to determine the necessary repeats to ensure reproducibility and statistical significance. The corresponding number of events that was analysed is indicated in the Figure legend or Methods section.
Data exclusions	No data were excluded from analysis.
Replication	We have indicated the number of independent experiments performed in the figure legends and additional information in the methods section.
Randomization	No randomization was performed for this study. Images were automatically acquired for the data analysis by high-content imaging. For super-resolution imaging the experimental setup included clearly defined conditions. To avoid bias, same software with identical settings between conditions was used for quantifications.
Blinding	No blinding was performed for this study. Blinding was not possible as all samples were analysed pairwise or multiple compared.

Reporting for specific materials, systems and methods

We require information from authors about some types of materials, experimental systems and methods used in many studies. Here, indicate whether each material, system or method listed is relevant to your study. If you are not sure if a list item applies to your research, read the appropriate section before selecting a response.

Materials & experimental systems

n/a	Involved in the study
☐	☒ Antibodies
☐	☒ Eukaryotic cell lines
☒	☐ Palaeontology and archaeology
☐	☒ Animals and other organisms
☒	☐ Clinical data
☒	☐ Dual use research of concern

Methods

n/a	Involved in the study
☒	☐ ChIP-seq
☒	☐ Flow cytometry
☒	☐ MRI-based neuroimaging

Antibodies

Antibodies used

Antibodies were: anti-G3BP1 (13057-2-AP) or alternatively, anti-G3BP1 (66486-1-Ig), anti-G3BP2 (16276-1-AP) anti-TIA1 (12133-2-AP), anti-PABPC1 (10970-1-AP), anti-ALIX (12422-1-AP), anti-CHMP2a (10477-1-AP), anti-CHMP4b (13683-1-AP), anti-Annexin A1 (66344-1-Ig), anti-Annexin A2 (66035-1-Ig), anti-EIF3B (10319-1-AP), and anti-EIF4G1 (15704-1-AP) from Proteintech. Anti-Galectin-3 (125410) and anti-Lamp1 (121610) from Biolegend. Anti-p62 (GTX111393) from GeneTex. Anti-PI4K2a (B-5, sc390026), anti-ORP9 (A-7, sc398961), and anti-G3BP1-546 (sc-365338 AF546) from Santa Cruz. Anti phospho-eIF2α (Ser51) (9721), anti- eIF2α (9722) and anti-β-Actin (8H10D10, 12262), and anti-phospho TBK1 (5483T) from Cell Signalling Technology; and HRP-conjugated anti-mouse (W4021) and anti-rabbit (W4011) antibodies from Promega.

Validation

All the antibodies purchased have been validated in multiple previous studies accessible at the manufacturer's website.
anti-G3BP1 (13057-2-AP), https://www.ptglab.com/products/G3BP1-Antibody-13057-2-AP.htm
anti-G3BP1 (66486-1-Ig), https://www.ptglab.com/products/G3BP-Antibody-66486-1-Ig.htm
anti-G3BP1 Alexa Fluor 546 (sc-365338 AF546) https://datasheets.scbt.com/sc-365338.pdf
anti-G3BP2 (16276-1-AP), https://www.ptglab.com/products/G3BP2-Antibody-16276-1-AP.htm
anti-TIA1 (12133-2-AP), https://www.ptglab.com/products/TIA1-Antibody-12133-2-AP.htm
anti-PABPC1 (10970-1-AP), https://www.ptglab.com/products/PABPC1,PABP-Antibody-10970-1-AP.htm
anti-ALIX (12422-1-AP), https://www.ptglab.com/products/PDCD6IP-Antibody-12422-1-AP.htm
anti-CHMP2a (10477-1-AP), https://www.ptglab.com/products/CHMP2A-Antibody-10477-1-AP.htm
anti-CHMP4b (13683-1-AP), https://www.ptglab.com/products/CHMP4B-Antibody-13683-1-AP.htm
anti-Annexin A1 (66344-1-Ig), https://www.ptglab.com/products/ANXA1-Antibody-66344-1-Ig.htm
anti-Annexin A2 (66035-1-Ig), https://www.ptglab.com/products/ANXA2-Antibody-66035-1-Ig.htm
anti-EIF3B (10319-1-AP), https://www.ptglab.com/products/EIF3B-Antibody-10319-1-AP.htm
anti-EIF4G1 (15704-1-AP), https://www.ptglab.com/products/EIF4G1-Antibody-15704-1-AP.htm
Anti-Galectin-3 (125410), https://www.biolegend.com/fr-lu/products/alexa-fluor-488-anti-mouse-human-mac-2-galectin-3-antibody-7084?GroupID=BLG2786
anti-Lamp1 (121610), https://www.biolegend.com/de-at/products/alexa-fluor-647-anti-mouse-cd107a-lamp-1-antibody-3589?GroupID=BLG4966
Anti-p62 (GTX111393), https://www.genetex.com/Product/Detail/SQSTM1-P62-antibody/GTX111393
anti-phospho TBK1 (5483T), https://www.cellsignal.com/products/primary-antibodies/phospho-tbk1-nak-ser172-d52c2-xp-rabbit-mab/5483?_requestid=3918912&gclid=Cj0KCQjwpc-oBhCGARIsAH6ote9PvI-e1E0t2t2P7d4LM9ghlrwW7TRzfpvqyiFjylYyxoaU8XrxsQsaAsFyEALw_wcB&gclsrc=aw.ds&_requestid=4404764
Anti-PI4K2a (B-5, sc390026), https://www.scbt.com/p/pi-4-kinase-ii-alpha-antibody-b-5
anti-ORP9 (A-7, sc398961), https://www.scbt.com/p/orp-9-antibody-a-7
phospho-eIF2α (Ser51) (9721), https://www.cellsignal.com/products/primary-antibodies/phospho-eif2a-ser51-antibody/9721
anti- eIF2α (9722), https://www.cellsignal.com/products/primary-antibodies/eif2a-antibody/9722
anti-β-Actin (8H10D10, 12262), https://www.cellsignal.com/products/antibody-conjugates/b-actin-8h10d10-mouse-mab-hrp-conjugate/12262#:~:text=Specificity%20%2F%20Sensitivity-,%CE%B2%2DActin%20(8H10D10)%20Mouse%20mAb%20(HRP%20Conjugate),react%20with%20cytoplasmic%20%CE%B3%2Dactin.
anti-mouse (W4021), https://www.promega.co.uk/products/protein-detection/primary-and-secondary-antibodies/anti_mouse-igg-h-and-l-hrp-conjugate/?catNum=W4021
anti-rabbit (W4011), https://www.promega.co.uk/products/protein-detection/primary-and-secondary-antibodies/anti-rabbit-igg-h-and-l-hrp-conjugate/?catNum=W4011

Eukaryotic cell lines

Policy information about cell lines and Sex and Gender in Research

Cell line source(s)

KOLF2 human iPSCs, Public Health England Culture Collections, Cat#77650100.
The use of human cells is covered and approved by the Ethical Committee and regulated by the Francis Crick Institute Biological Safety Code of Practice in the project registered at the Crick (Project HTA17) framed under Human Tissue Authority Licence number 12650
HeLa cells: Cell Services, The Francis Crick Institute.
U2OS WT and G3BP DKO cells: Paul Anderson Laboratory (Harvard University), 10.1083/jcb.201508028
mEGFP-G3BP1 human iPSCs, Coriell Institute (AICS-0082-001)

Authentication

Authentication results for human mEGFP-G3BP1 iPSCs can be accessed at https://catalog.coriell.org/0/Sections/Search/Sample_Detail.aspx?Ref=AICS-0082-001&Product=CC Authentication results for KOLF2 human iPSCs can be accessed at the respective source's website. https://www.phe-culturecollections.org.uk/products/celllines/generalcell/search.jsp KOLF2

	hiPSC are routinely authenticated at the lab by flow cytometry. Cell line authentication was initially performed by ATCC. Further authentication (U2OS, HeLa) was performed by microscopy at our lab and at The Francis Crick Cell Services unit.
Mycoplasma contamination	All cells tested negative for mycoplasma contamination.
Commonly misidentified lines (See ICLAC register)	No ICLAC cell lines were used in this study.

Animals and other research organisms

Policy information about studies involving animals; ARRIVE guidelines recommended for reporting animal research, and Sex and Gender in Research

Laboratory animals	Six- to eight-week-old, C3HeB/FeJ mice were used in this study. All mice were maintained in BSL3 cages, at 22°C ± 2°C and a relative humidity of 55 ± 10%.
Wild animals	No wild animals were used in this study.
Reporting on sex	ARRIVE guidelines and previous studies (10.1016/j.chom.2017.04.004) were followed to define animal cohorts. 5 animals per group were used per time of infection. Females were used for safety and space allocation restrictions as infected mice were contained in BSL3.
Field-collected samples	No field collected samples were used in this study.
Ethics oversight	All protocols for breeding and experiments were approved by the Home Office (U.K.) under project license P4D8F6075 and performed in accordance with the Animal Scientific Procedures Act, 1986.

Note that full information on the approval of the study protocol must also be provided in the manuscript.
