# Supplementary material for: Stress granules plug and stabilize damaged endolysosomal membranes
Source: Nature. 2023 Nov 15;623(7989):1062–9. doi: 10.1038/s41586-023-06726-w (PMC10686833; doi:10.1038/s41586-023-06726-w)

Blots related to Figure 4b

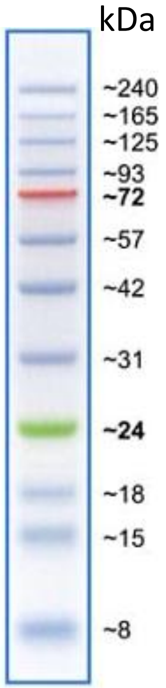

Prestained Protein Ladder – Broad molecular weight (10-245 kDa) (ab116028)

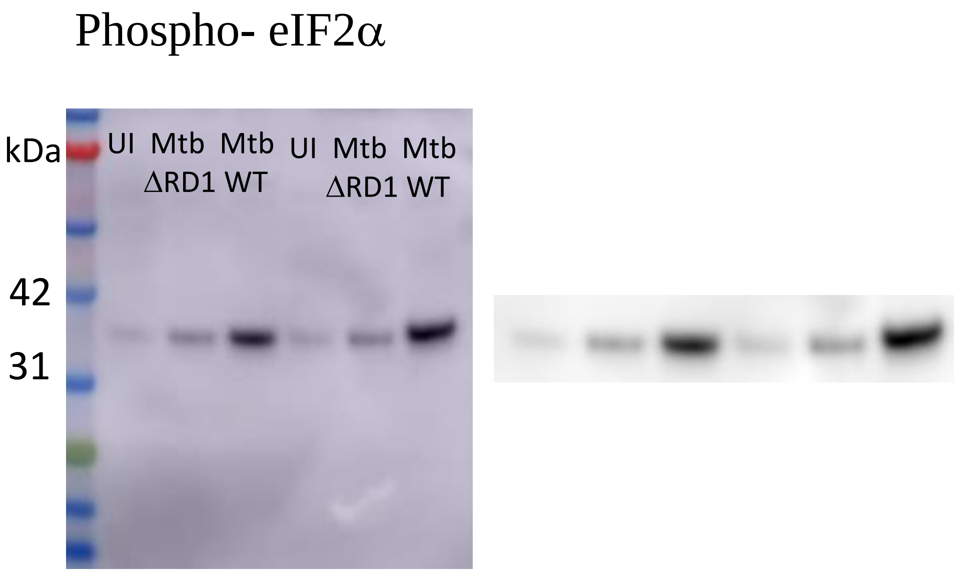

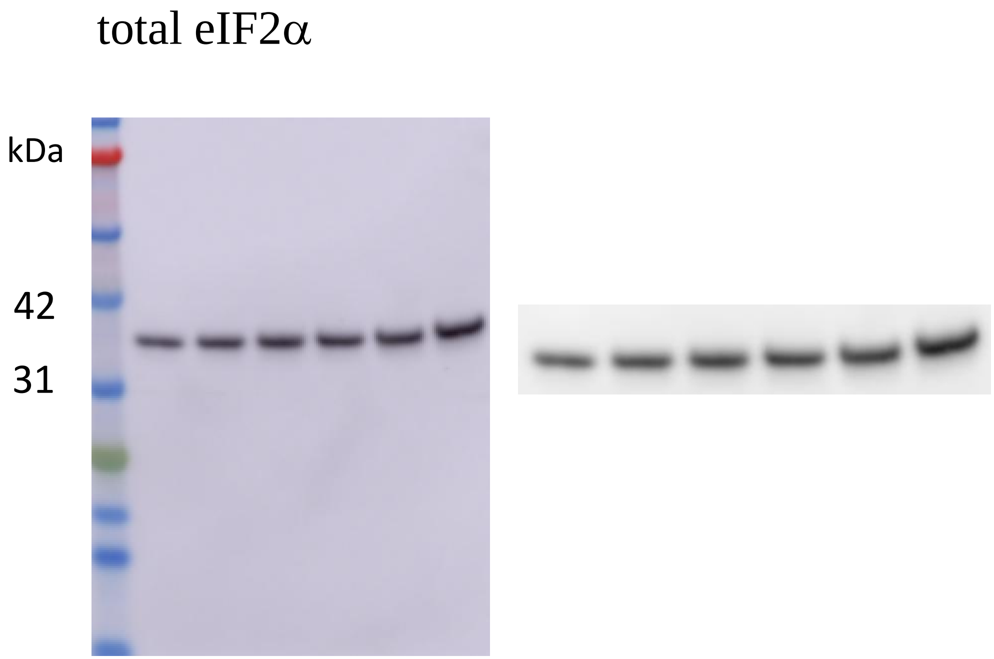

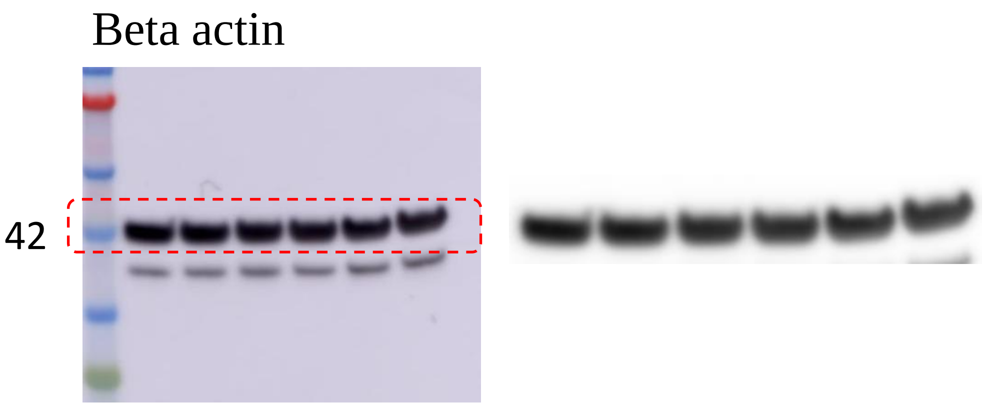

Blots related to Extended Data 2c

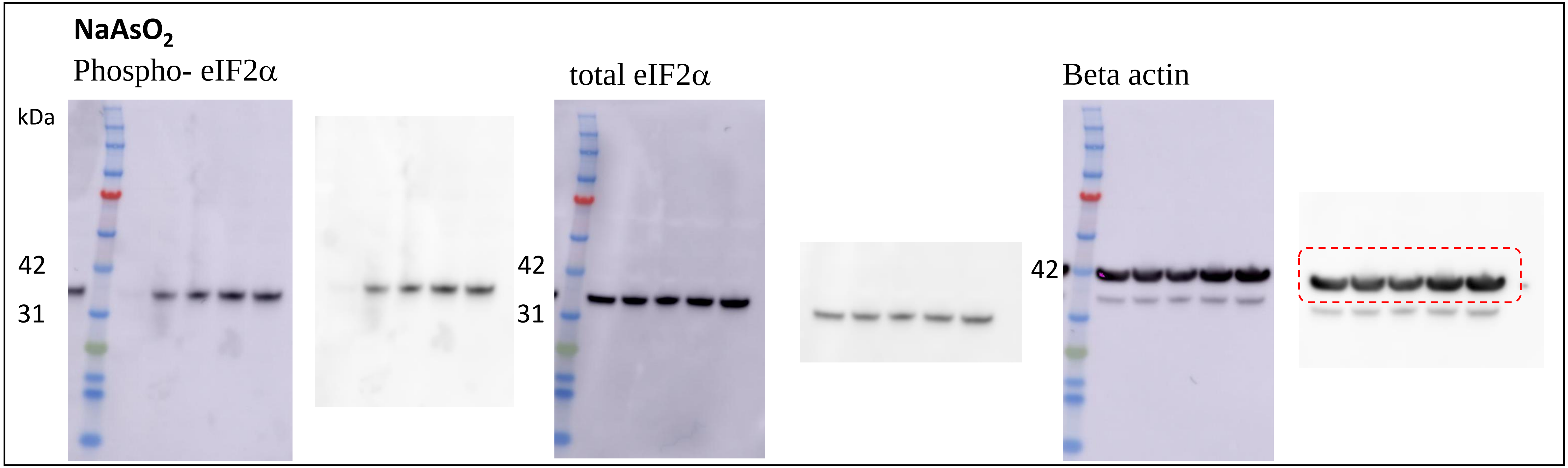

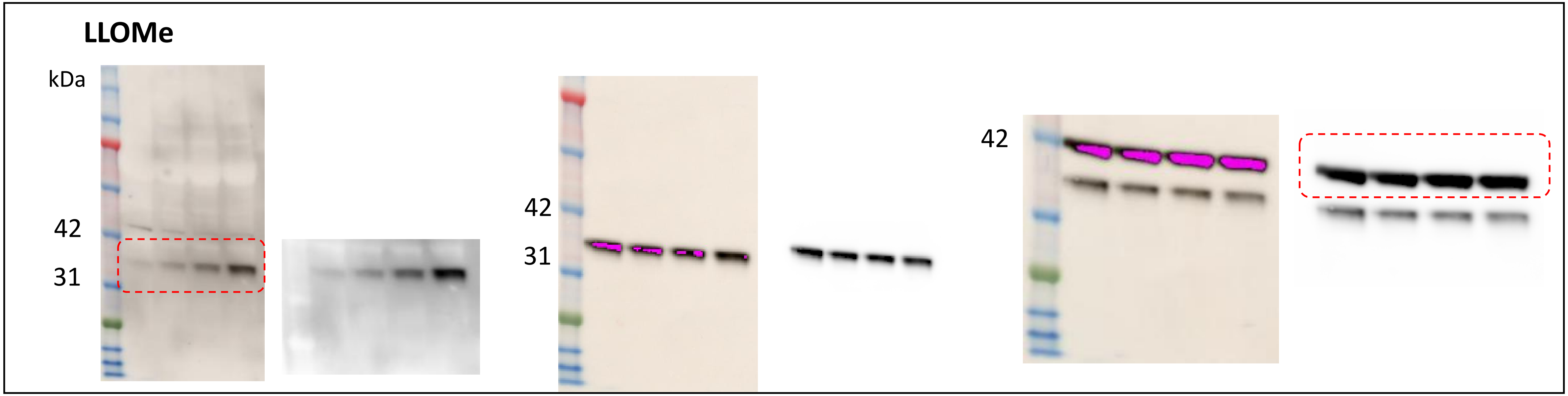

Blots related to Extended Data 6b,h,i

Extended Data 6b
(iPSDM)

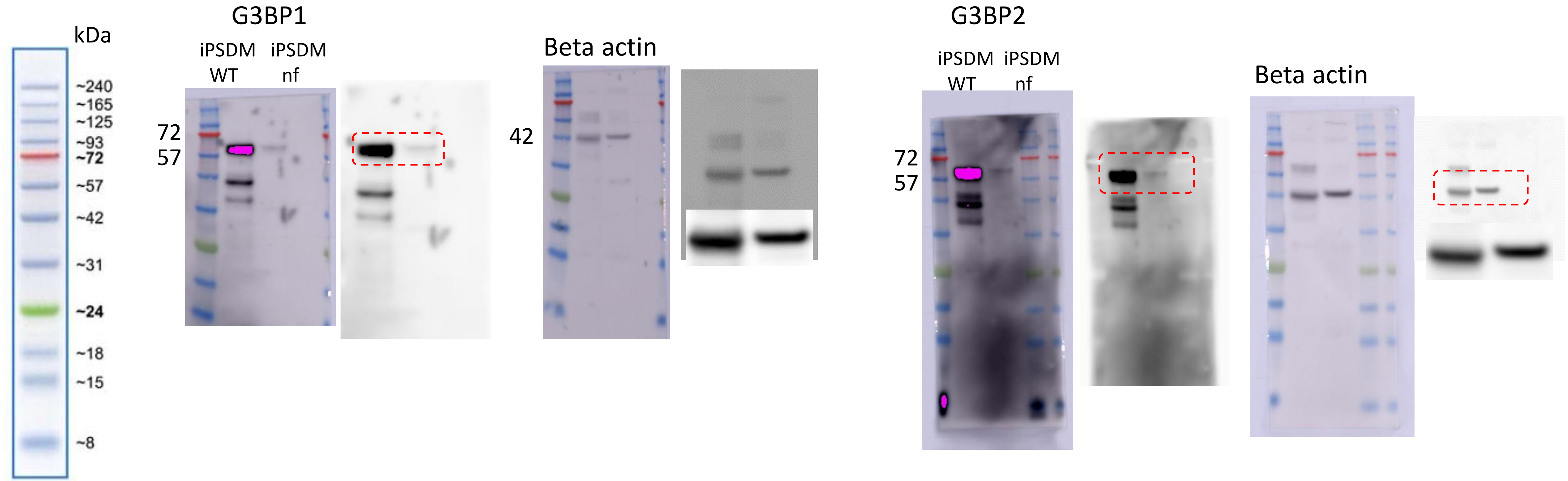

Prestained Protein Ladder – Broad molecular weight (10-245 kDa) (ab116028)

Extended Data 6h
(HeLa cells)

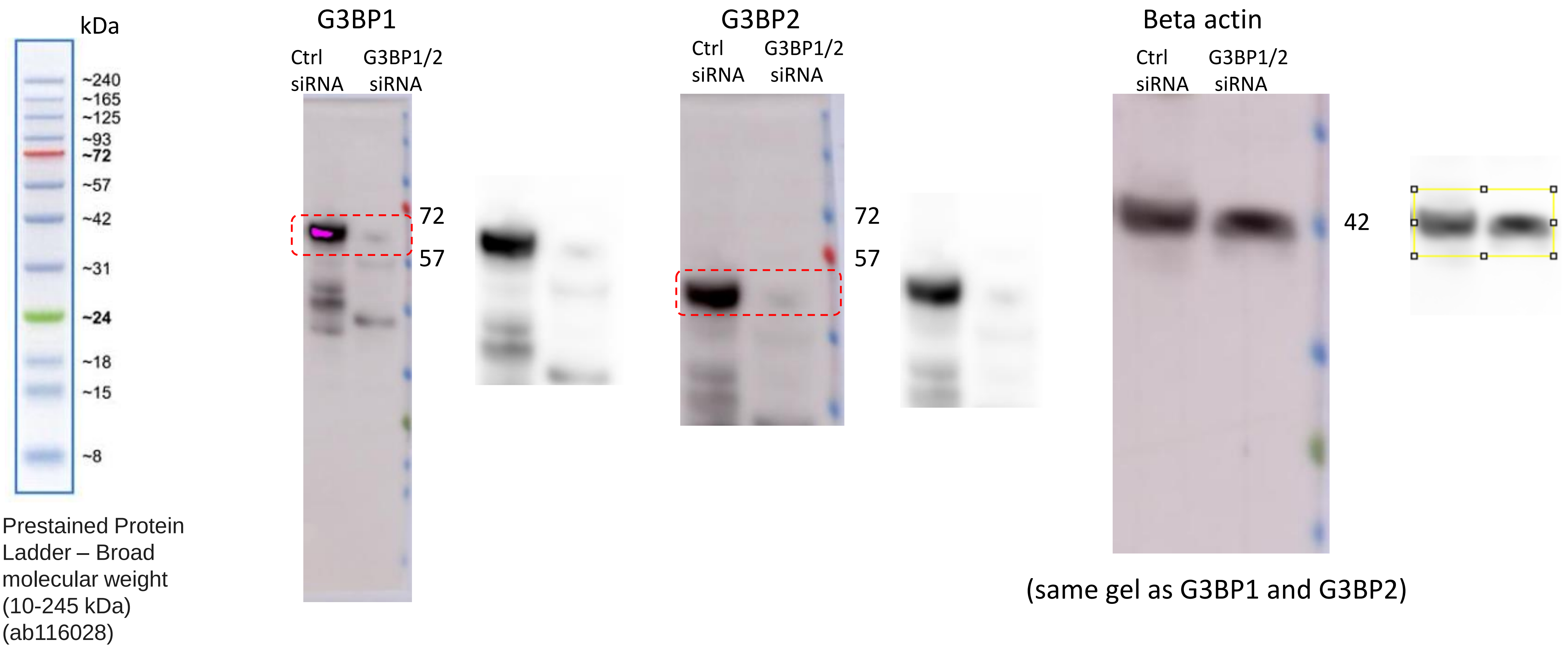

Extended Data 6i (hMDM)

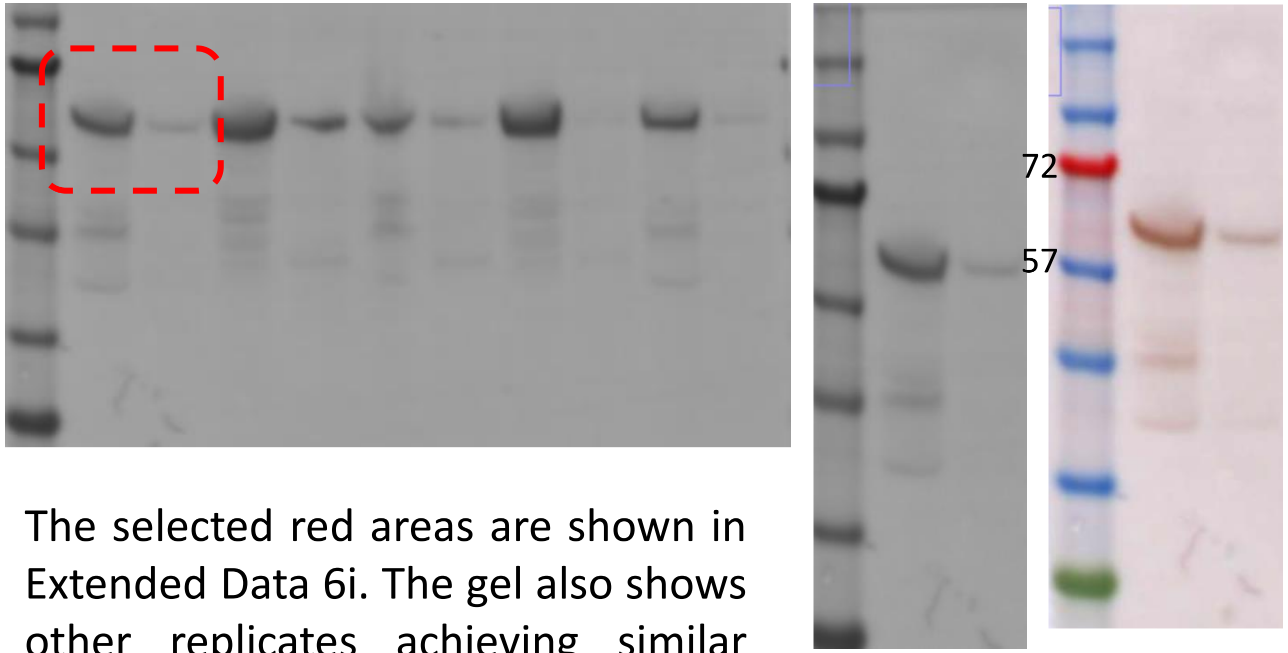

The selected red areas are shown in Extended Data 6i. The gel also shows other replicates achieving similar results (1st line WT, 2nd line nucleofected cells).

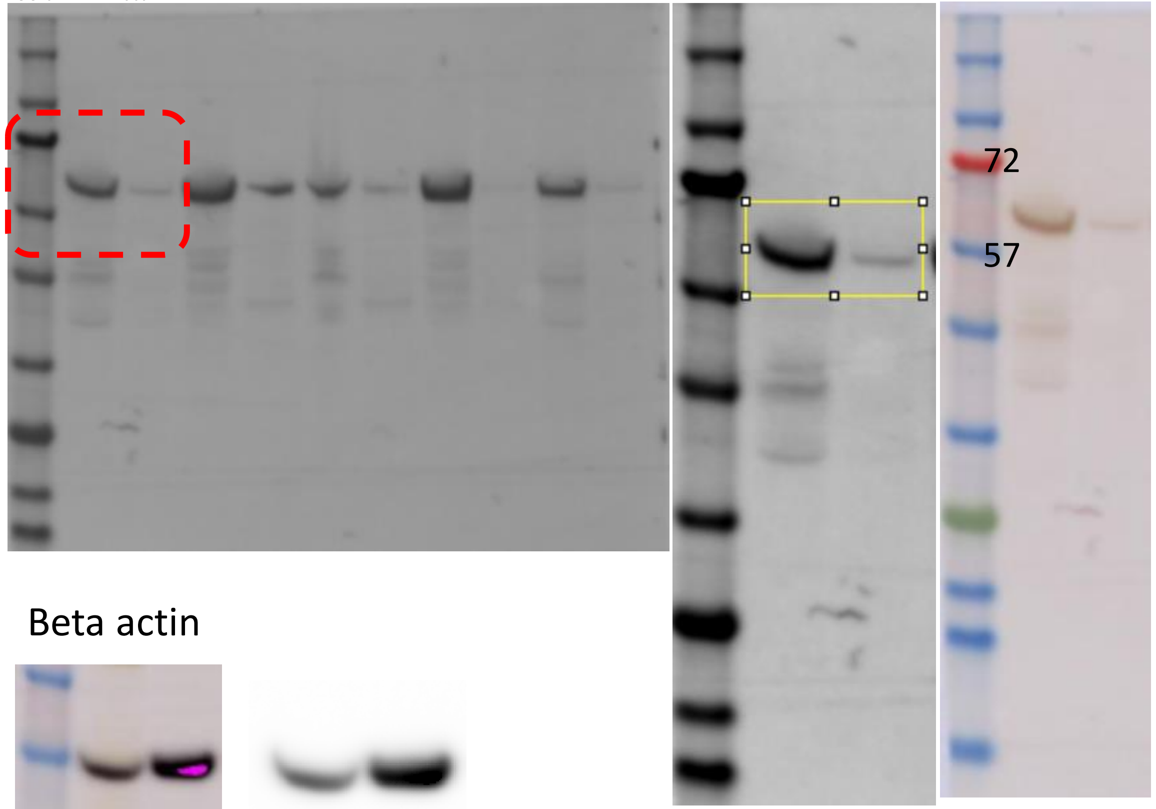

Beta actin

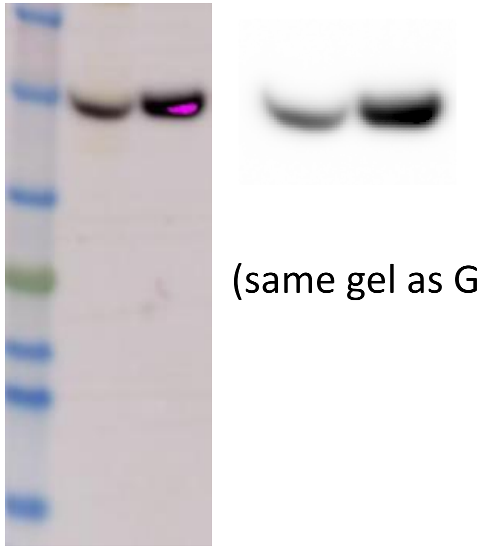

(same gel as G3BP1 and G3BP2)

Blots related to Extended Data 7c

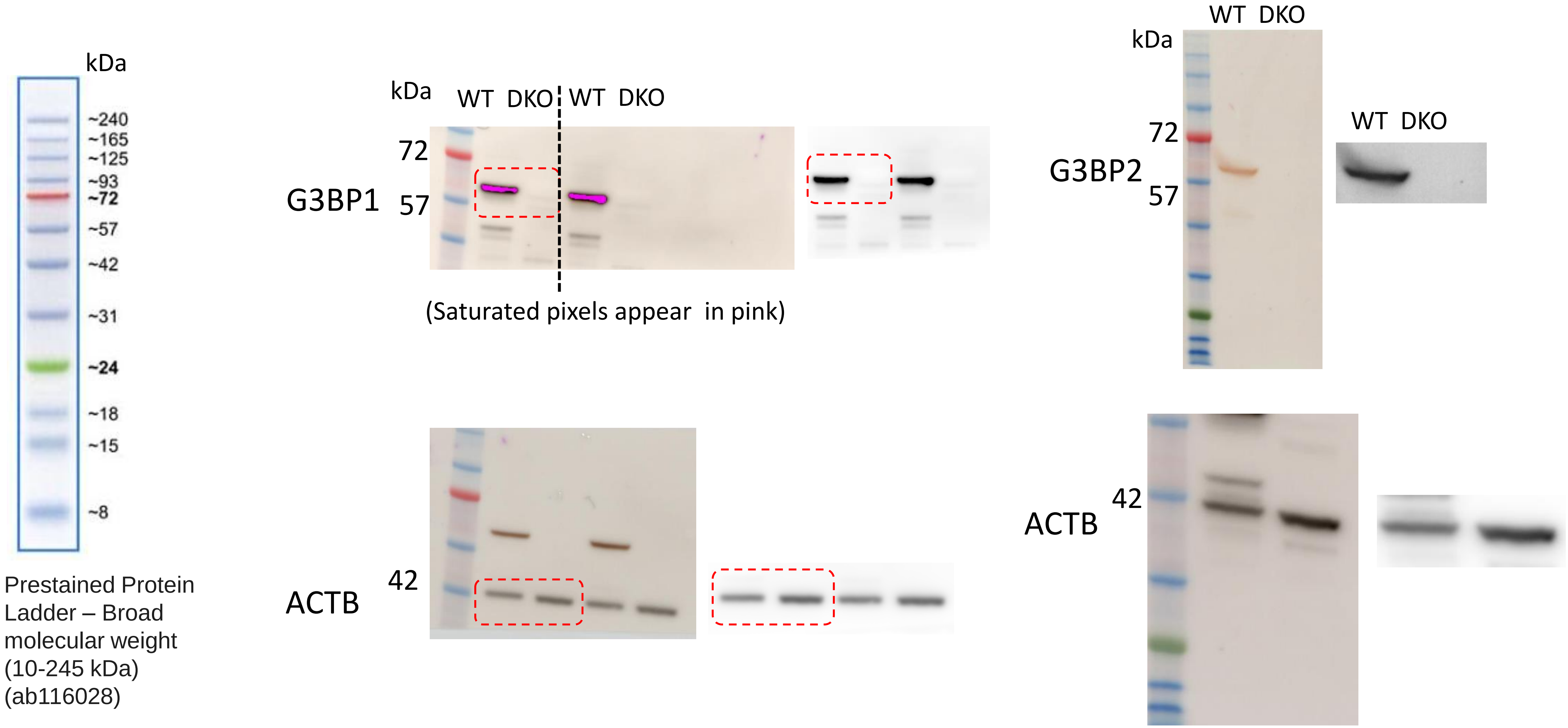

Supplement: Supplementary file 3 — Uncropped western blot images. [file 41586_2023_6726_MOESM3_ESM.pdf]
